# Design and development of a content-based medical image retrieval system for spine vertebrae irregularity

**DOI:** 10.1186/1475-925X-14-6

**Published:** 2015-01-16

**Authors:** Aouache Mustapha, Aini Hussain, Salina Abdul Samad, Mohd Asyraf Zulkifley, Wan Mimi Diyana Wan Zaki, Hamzaini Abdul Hamid

**Affiliations:** Department of Electrical, Electronic and Systems Engineering, Faculty of Engineering and Built Environment, Univeristi Kebangsaan Malaysia, Bangi, 43600 Selangor DE, Malaysia; Department of Radiology, Faculty of Medicine, Univeristi Kebangsaan Malaysia Medical Centre, Jalan Yaacob Latif, Bandar Tun Razak, 56000 Kuala Lumpur, Malaysia

**Keywords:** X-ray images, Vertebrae irregularity, Modelling, Indexing, Retrieval, CBMIR

## Abstract

**Background:**

Content-based medical image retrieval (CBMIR) system enables medical practitioners to perform fast diagnosis through quantitative assessment of the visual information of various modalities.

**Methods:**

In this paper, a more robust CBMIR system that deals with both cervical and lumbar vertebrae irregularity is afforded. It comprises three main phases, namely modelling, indexing and retrieval of the vertebrae image. The main tasks in the modelling phase are to improve and enhance the visibility of the x-ray image for better segmentation results using active shape model (ASM). The segmented vertebral fractures are then characterized in the indexing phase using region-based fracture characterization (RB-FC) and contour-based fracture characterization (CB-FC). Upon a query, the characterized features are compared to the query image. Effectiveness of the retrieval phase is determined by its retrieval, thus, we propose an integration of the predictor model based cross validation neural network (PMCVNN) and similarity matching (SM) in this stage. The PMCVNN task is to identify the correct vertebral irregularity class through classification allowing the SM process to be more efficient. Retrieval performance between the proposed and the standard retrieval architectures are then compared using retrieval precision (*Pr@M*) and average group score (*A*_*GS*_) measures.

**Results:**

Experimental results show that the new integrated retrieval architecture performs better than those of the standard CBMIR architecture with retrieval results of cervical (*A*_*GS*_ > 87%) and lumbar (*A*_*GS*_ > 82%) datasets.

**Conclusions:**

The proposed CBMIR architecture shows encouraging results with high *Pr@M* accuracy. As a result, images from the same visualization class are returned for further used by the medical personnel.

## Background

### Justification for research

Vertebral irregularity (VI) or fracture is an extremely familiar complication of anterior osteoporosis (AOs) that has become a major public health concern. An early intervention of VI is very essential because a timely pharmalogical involvement can be applied to decrease the risk of further vertebral fracture. Although a vertebral fracture is visible on lateral x-ray radiograph but investigators have noted that it is frequently undetected by clinicians [1] and under diagnosed by radiologists [2] even when the fracture is severe. Therefore, the primary objective of this research is to develop a computerized system for retrieving and screening cervical and lumbar vertebrae images to facilitate VI diagnosis. Our eventual tasks to achieve this objective are : (i) to acquire and annotate a large cervical and lumbar database image, (ii) to examine and implement a modelling algorithm which can automatically locate all vertebrae, (iii) to evaluate the algorithm capacity in retrieving the vertebrae shape that may or may not contain VI and (iv) to develop a computerized diagnostic tool for clinicians use in large-scale clinical trials and epidemiological studies. Two major issues have been considered and regarded as significant challenges in the development of such CBMIR system.

First issue relates to the examination of practical algorithms for vertebral irregularity assessment, which is typically affected by (i) the inferior quality of x-ray images that results in poor segmentation and prone to mix up tissue and vertebra boundaries [3], and (ii) the region of interest (ROI) variations since typically, the vertebrae has a wide range of shape, size, and orientation. As such, the determination of vertebrae shape boundary is difficult to dissect correctly. The second issue relates to the CBIMR design and architecture. The proposed CBMIR system aims to accommodate various needs of medical practitioners and researchers. For example, a member of a medical team, who specializes in identifying spinal fracture and disease, should be able to query previous cases of slight anterior osteoporosis (AO) of the cervical spine for both genders. Likewise, a clinician can use this system to search an image that is similar to the patient condition who has presented the same pathology. In addition, the proposed system is hoped to be beneficial for medical research, education, clinical trials, other diagnostic applications etc.

This paper is organized as follows: it begins with a section on "Background" that reviews related researches and prototypes, followed by a section on "Vertebrae irregularity description" that defines and describes the bone irregularities considered in this work, which is AOs in both cervical and lumbar x-ray image. Subsequently, the section on "CBMIR system design and development" describes the design framework, the three main phases involved in the development of the CBMIR system and the developed "CBMIR software tool". Results and analysis are presented in the "Results and discussion" section and the paper concludes with a section named as "Conclusion".

### Literature review of related research

Several functional research prototypes of (CBMIR) have been designed in the last decade [4, 5], while a few systems are still in the development phase with a strong focus on database and indexing procedures. Some examples of prominent prototypes are as follows:

The ASSERT system [4] which is a Physician-in-the-Loop CBIR System designed for High Resolution Computed Tomography (HRCT) Image Database. It is proposed for the diagnosis of the lung condition that employs HRCT images. This system utilizes a set of features that determine the anatomical perceptual properties such as nodular, linear and reticular opacities as well as high and low spread of the shrinking regions. Some properties that can be inferred from these features are, total cell count and average dimensions, thickness of bronchi wall and number of cells neighbouring to the lung limitations bounder or crakes etc.

CBIR2 [6] is a prototype system that deals with spine x-ray radiography retrieval. The indexing method comprises algorithm for segmentation, feature computation and organization of text data. This prototype utilizes twenty-five of biomedical features based on the region of interest in the spine radiograph images. Even so, only three biomedical features is usually regularly diagnosed by human perception based on feature indexing, which are Anterior Osteophyte (AO), Disk Space Narrowing (DSN) as well as subluxation and spondylolisthesis for both cervical and lumbar spine. CBIR2 system is in fact an extension of the prior text based WebMIRS system [7].

Pathfinder [8] is CBIR system based wavelet transform, which preserves spatial information of the image. CBIR system based on wavelet transform is found to be suitable and appropriate for the medical imaging field. It has been demonstrated to be successful in searching and retrieving of medical images of pathology slides. Image Retrieval in Medical Application or simply IRMA [9] is an application system that combines Picture Archival and Communication Systems (PACS) and CBIR to build a comprehensive diagnostic verification dependent medication and event dependent reasoning. The key idea of IRMA system is based on six-step process; image (i) categorization and (ii) registration, (iii) feature extraction, (iv) multi-scale indexing, (v) identification, and (vi) retrieval.

I-BROWSE [5] is considered as smart content-based indexing and browsing of medical images. The primary target in this system is usually to support intelligent searching and retrieval of histological images from the gastrointestinal tract, in which high level semantic features are archived and textual annotations are auto-generated using knowledge bases and reasoning engines.

In recent years, research in CBIR algorithms [10] has attracted a large interest, particularly in the indexing of biomedical images. Kuo et al. [11] has used breast sonogram to automatically detect any potential breast disease. The data set was indexed manually by locating regions of interest (ROIs) that will be the basis for future generation. After the images have been indexed, it will be presented to the CBIR system to ensure a status of similar images might be identified as a result of K-top similar images while using texture in addition to weighted Euclidean distance.

Besides that, to facilitate radiologists like double reading, [12] propose a computer-aided diagnosis (CAD) system for breast micro-calcifications predicated on dual-tree complex wavelet transform (DT-CWT). Not like standard wavelets, DT-CWT could possibly summarize the features better, and the proposed CAD program received some sort of aggressive overall performance. Moreover, [13] propose a new content-based search for dermoscopic photographs to support specialized medical decision making. The system can locate, retrieve along with screening dermoscopic images identical to a minimum of one that is given as a query by using some simple capabilities definitely not linked to any kind of specific diagnostic that are capable to visually define the image.

An innovative partial shape matching (PSM) technique using dynamic programming (DP) for the retrieval of spine X-ray images proposed by [14] was reported to have high precision. It used boundary points for DP search and improved matching speed by approximately 10 times compared to traditional DP. The method is invariant to translation, scaling, rotation, and the starting point selection. The retrieval accuracy and processing speed of the retrieval system, were based on the new corner-guided PSM method. Another CBIR that used both the 9-point model, familiar to radiologists and bone morphometrists, and the computationally meaningful 36-point vertebral shape profiles was described in [15]. The work had used feature ranking and voting consensus and was able to improve retrieval accuracy by 8.25%.

The problem of indexing shapes in medical image databases was addressed by [16]. They proposed an indexing technique by embedding optimal finite shape sets from a shape space into a Euclidean space to retrieve images of the vertebral shape from the NHANES II. Experimentally, the work concluded the importance of shape indexing and the advantage of using their proposed technique.

Radiology images pose specific challenges compared with images in the consumer domain; but they contain varied, rich, and often subtle features that need to be recognized in assessing image similarity. As stated in [17], radiographs comprised rich meta-data about image semantics provided by radiologists and therefore, provide rich opportunities for CBIR that has not being used to its fullest advantage.

A recent survey by [18] described the state-of-the-art medical CBIR studies that have been applied in the retrieval of 2D images, images with multiple dimensions, and multimodality images from repositories containing a diverse collection of medical data. They noted that although CBIR systems have evolved from 2D image retrieval to multidimensional and multimodality image retrieval, there still remain several challenges to tackle particularly, those that relate to retrieval visualization and interpretation, feature selection from multiple modalities, efficient image processing, and development of retrieval algorithms and systems for clinical applications. Further investigations in the aforementioned areas should be pursued to produce CBIR frameworks that are practical, usable, and most importantly, have a positive impact on healthcare.

As summarized above, the problem of medical imaging diagnosis (MID) systems has been widely studied in the past years. However, they still represent a difficult research area especially in regards to locating regions of interest (ROIs), automated image indexing, classification, and content-based image retrieval. Furthermore, the necessity for further investigation is strongly encouraged to abide the needs and requirements of medical practitioners and researchers.

### Contribution

A new CBMIR architecture of spine x-ray image indexing and retrieval for vertebrae irregularity specifically, AOs has been proposed and developed. The new architecture focuses on the retrieval phase which involves integration of the PMCVNN and SM thus allowing a more intelligent approach to improve the retrieval performance. In this work, several fracture characterization algorithms, Gabor Wavelets (GW), Gray Level Co-occurrence Matrix (GLCM), Radon Transform (RT), Orientation Histogram (OH) and Fourier Descriptors (FDs) based on two different regions of interest (ROIs), namely the 9APR and BSR, were investigated and evaluated. In addition, a new GUI has been created to facilitate the end-user to visualize similar images of their queries for medical related applications.

## Vertebrae irregularity description

### AOs in cervical vertebrae

Two schemes have been considered for the classification of AOs fracture in cervical vertebra and the schemes are Macnab’s classification (MC) [19, 20] and severity grading (SG) system [21, 22]. The MC scheme acknowledges three classes of AOs, which are claw, traction and claw-traction. Their visual characteristics are as follows; for the claw spur, it often has a triangular shape curve at the tip that increases through the vertebral rim and curves to the surrounding disk whereas for the traction spur, it has moderate thickness that protrudes the adjacent disk horizontally and does not curve at the tip.

**Figure 1:**
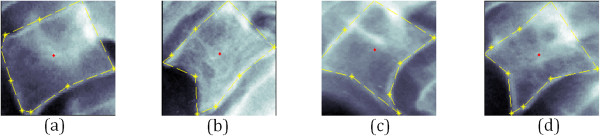
**Examples of AOs classes of the cervical x-ray images.** Samples of Macnab’s classification and their osteophyte severity grading in the cervical vertebrae x-ray images, **(a)** normal **(b)** moderate traction **(c)** severe claw and **(d)** slight claw-traction.

Consequently, the SG system is a scheme which is defined by the medical experts based on reasonable criteria by manually assigning the severity level of the AOs. There are three severity levels of AOs [21, 22] defined by the scheme which are slight, moderate, and severe cases. By combining both of MC and SG classification schemes, nine different kinds of pathology are obtained, namely slight claw, moderate claw, severe claw, slight traction, moderate traction, severe traction, slight claw-traction, moderate claw-traction and severe claw-traction. In this work, a total of 10 classes were used which include all nine above mentioned classes and the normal class. Figure 1 illustrates selected examples of cervical x-ray images which are a) normal and three different pathologies of vertebrae irregularities; b) moderate traction c) severe claw and d) slight claw-traction.

### AOs in lumbar vertebrae

**Figure 2:**
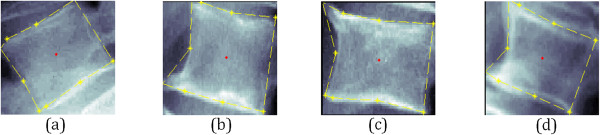
**Examples of AOs classes of the lumbar x-ray images.** The four classes of AOs in lumbar vertebrae **(a)** normal **(b)** L-LHC **(c)** U-LHC and **(d)** B-LHC.

The AOs in lumbar vertebrae focus on the vertebral distortion across the anterior boundary [23]. Figure 2 displays selected examples of lumbar x-ray images which are a) normal and three different pathologies of vertebrae irregularities; (b) osteophytes in the lower left-hand corner (L-LHC), (c) osteophytes in the upper left-hand corner (U-LHC), and (d) osteophytes in both left-hand corners (B-LHC).

## CBMIR system design and development

### Description of the design framework

**Figure 3:**
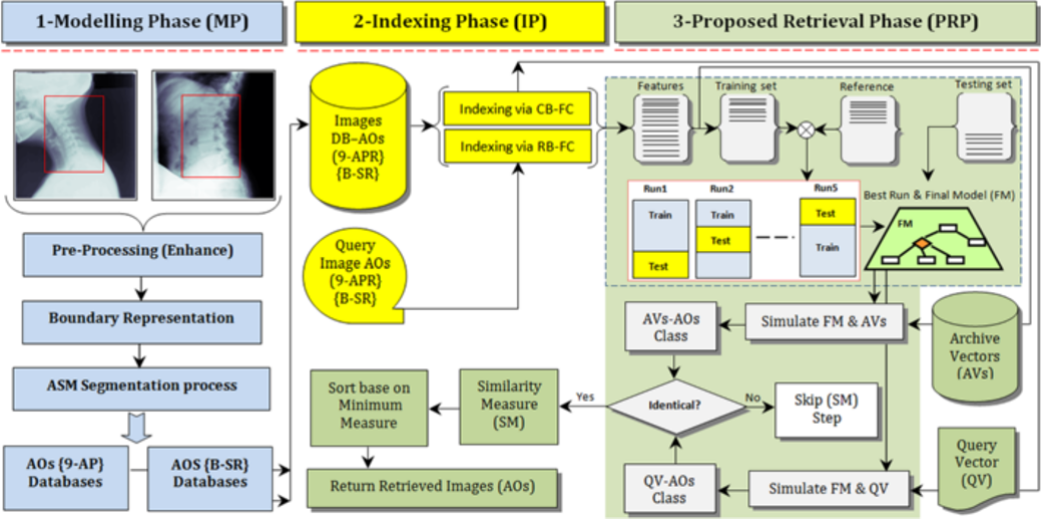
**Main phases involved in developing the proposed CBMIR for spine vertebrae irregularity assessment.** The region localization (RL) of the cervical and lumbar x-ray images are presented within the red boxes, RL-Cervical (C3-C6) and RL-Lumbar (L1-L5). From left-to-right, the blue coloured diagram signifies the MP steps. The diagram in yellow represents the IP and green represents the PA for the retrieval phase.

The design framework for the development of the proposed CBMIR system for AOs vertebrae irregularity involves three main phases that are, the Modelling Phase (MP), Indexing Phase (IP) and The Proposed Architecture (PA) for the retrieval phase. The MP comprises the pre-processing and segmentation algorithms using active shape model (ASM) involving the 9-anatomical point representation (9-APR) and B-spline representation (B-SR) to extract the vertebrae shape. The IP deals with implementation of two different approaches of fracture characterization to extract features of the vertebrae shape or boundary. The approaches are region based fracture characterization (RB-FC) and contour based fracture characterization (CB-FC). Finally, the PA for the retrieval phase involves integration of PMCVNN, to identify the correct vertebral irregularity class through classification, and the SM, to measure and rank the resulting classified image features. Figure 3 shows the main phases in the CBMIR design framework. The detail descriptions of the three main development phases are described in the subsequent sub-sections.

### Description of the development phases

This section affords the details of the three main phases involved in developing the CBMIR system. On top of that, another important aspect that need to be considered during the development of such system, is the establishment of the ground truth for all classes of irregularity been studied in this work. Consequently, this section described the main phases of the development process and also briefly mentioned the establishment of ground truth.

#### The modelling phase (MP)

**a) Pre-processing:** X-ray images are typically corrupted by additive noise, which results in poor quality images that have low contrast with very minimal information concerning on the pathologies of interest to the medical researchers. De-noising method can improve and enhance the visibility of some bone structures in the image. So as to produce satisfactory visual information for the radiologists, the x-ray images quality need to be enhanced by noise reduction. In this work, the pre-processing approach using adaptive factors based on non-linear contrast adjustment, which was proven to be effective by [3], was adopted to reduce the noise in cervical and lumbar radiographs.

**Figure 4:**
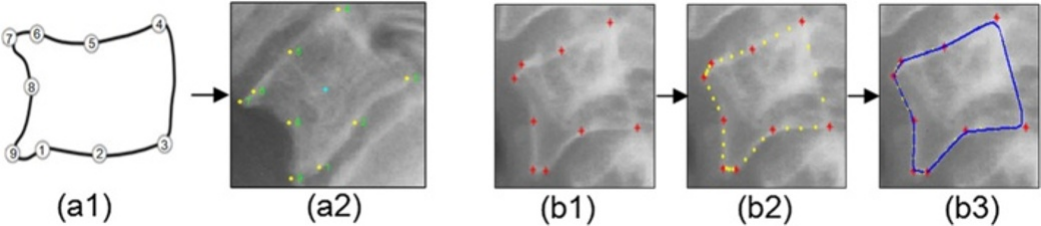
**Shape boundary representation of the 9-APR and the third order B-spline schemes.** The 9-anatomical points are marked by expert radiologist, Figure 4 **(a)** represents the marked made on **(a1)** synthetic and **(a2)** authentic vertebra image. Figure 4 **(b)** depicts the generated shape boundary using the 3rd order B-SR in which **(b1)** represents the 9-APR points, **(b2)** depicts the 27 equally spaced point over the boundaries measured from (b1) and **(b3)** depicts the resulting boundary of the vertebra using B-SR.

**b) Shape boundary representation:** The AO is a pathological characteristic which can be dependably and persistently recognized along the vertebral border seeing from the vertebra sagittal view. To be more specific, it is typically discovered down the anterior superior or the inferior edges. As defined by [14] using 9 anatomical points representation (9-APR), the four corners of the vertebral shape are indicated by points 1, 3, 4, 6, in which points 3 and 4 indicate the lower and upper posterior corners of the vertebral shape respectively. Accordingly, points 1 and 6 indicate the lower and upper anterior corners of the vertebral body, respectively whereas points 2 and 5 indicate the median location across the lower and upper vertebra shape. Point 8 is the midpoint along the anterior vertical shape. Points 7 and 9 mark the upper and lower AOs, but if these points coincide and overlap with the points 6 and 1, respectively, then it can be concluded that AOs are not presence on the vertebrae image. Figure 4 (a1, a2) depicts the 9-APR scheme.

Since the bone shape varies from one person to another, the manual 9-anatomical point’s placement may not be a suitable representation. Therefore, the B-spline representation (B-SR) of the third order by De Boor [24] is adopted to produce a better shape boundary [3]. In this work, the B-SR representation has been implemented successfully to robustly obtain a complete boundary vertebra shape in the lateral vertebra x-ray image as shown in Figure 4 (b1, b2, b3).

**Figure 5:**
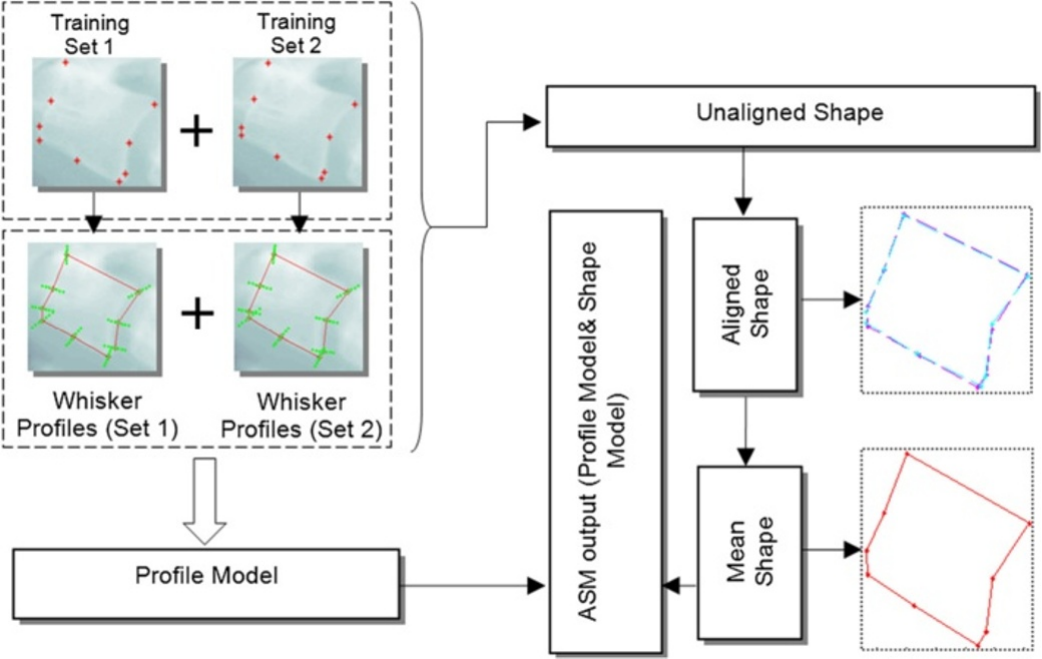
**Summary of the ASM training process using the 9-APR.**

**c) Active shape model (ASM) segmentation process:** The ASM, proposed by [25], has to be trained using training images where the contours of the cervical and lumbar vertebrae are characterized by a set of landmark points. It requires good initialization points of the vertebrae pose to produce an accurate segmentation. The first step in constructing the ASM is to create a statistical model of the vertebrae appearance via a collection of annotated training samples. The shape boundary serves two purposes, which are to provide a rich description of the vertebra shape and edges that are visually acceptable to the medical researchers. A set of appropriate landmark points that summarize the vertebrae shape boundary must be determined before the spinal vertebra model can be constructed. Therefore, the two shape boundary representations, as mentioned above, have been used as the foundation for ASM training stage.Figure 5 graphically summarizes the ASM segmentation training process that includes unaligned shape from the training images, aligned shapes after pre-processing and the whisker profiles. The landmark points are shown as red dots, representing the sample points used during the training stage of the 9-APR scheme. The magenta, blue and red coloured outlines, shown on the right hand side, indicate the two sets of aligned shape and its mean shape, respectively. Referring to the left hand side of Figure 5, the whisker profiles, shown in green, are the superimposed shape of the vertebrae x-ray image. The points that are perpendicular to the model are called "whiskers", and they are useful in analysing the areas around the marked points. The ASM produces two sub-models, known as the profile and shape model, which are obtained after the vertebra has been landmarked and undergo the full process for shape alignment. The sub-models are described as follows.

i) Profile Model: This model is used to analyse the landmark points and then stores behaviour information of the image around the landmark points by learning their characteristics during training, in order to build a profile model for each landmark point. During searching for the shape in the test image, the region nearby the tentative landmarks is examined and the tentative landmarks are adjusted accordingly, to the location that fits closely to the profile model.
ii) Shape Model: This model represents the acceptable relative positions of landmarks. It ensures that the mean shape does not change while the profile model tries to adjust and match the model in the test image. The profile model acts on individual landmarks, whereas the shape model acts on the image globally. Both models work by correcting each other until no further improvement is possible.

Upon completion of the training process, the collected information details that best fit the model towards the target has to be determined to delineate an object using the model. The test image is searched for the vertebrae shape by superimposing the mean shape calculated during training and by examining the profiles around the landmark points.

#### The indexing phase (IP)

The IP involves various feature extraction algorithms of the segmented shapes obtained previously. In this work, several feature extraction methods to characterize fraction based on region and contour approaches have been investigated and described as follows.

**a) Region-based fracture characterization (RB-FC) approach** In the RB-FC approach, all pixels within the images are taken into consideration while extracting the N-dimensional feature vector of AOs. In this work, a new method to produce the skeleton images that serve as basis for feature extraction was developed via the combine use of Euclidean distance transform (EDT), watershed transform and Radon transform (RT). In this section, the RB-FC approach recognizes and describes the vertebrae irregularity using four different feature extraction algorithms, namely Gabor wavelet (GW), Grey Level Co-Occurrence Matrix (GLCM), Radon Transform (RT) and Orientation Histogram (OH).

i) GW- The GW filter-bank was employed to extract and characterize features from the resulting (EDT) images presented with or without fracture using Equation (1) developed by [26]. 1

Where, , *j* = 0,1,2.. and *θ* ∈ [0,2*π*] and the particular different choices of frequency *j* along with its orientation *θ* are used to create a set of filters. An analysis was conducted using various frequency levels and mask sizes so as to pick the optimized filter-bank coefficients. The optimized filter-bank can then be used to distinguish between AOs fractures efficiently. The elementary GW function with the optimized filter bank is employed to develop the spatial domain filters consisting of real and imaginary parts of the complex sinusoid. This paired filter convolved and assembled with the EDT obtained from the AO images using both the 9-APR and B-SR. For each frequency level, the GW normalized filtering vector points ( ) denotes the average convolution from the real  and imaginary  filter masks computed as follows :
2

ii) GLCM- The GLCM is a matrix where the row number as well as column is usually equal to the grey levels (*G*) number in the image. The particular GLCM component *D*(*i*,*j*|*Δ*_*x*_,*Δ*_*y*_) is the relative frequency, along with a pair of pixels, segregated by the pixel distance (*Δ*_*x*_,*Δ*_*y*_) which occur within a given neighbourhood with intensity (*i, j*) provided that the *M* × *N* neighborhood of EDT input image contain *G* gray levels ranges from 0 to *G-1*. Letting the *EDT(m,n)* be the intensity at sample (*m*), line (*n*) of the neighborhood. Then
3

where
456

Fourteen textural features extracted using the GLCM were introduced in the classical work [27] which involved massive computation. As such, to reduce the computational complexity, as suggested by [28], only six textural features were considered instead of all fourteen. The common statistics applied to co-occurrence probabilities are: Contrast (Cn), Energy (En), Entropy (Et), Homogeneity (Ho), Correlation (Co), Variance (Va) and these features are considered to be the most appropriate features to represent the statistical features derived from the GLCM computation load (*f*_*GLCM*_) given as:
7

and the GLCM normalized vector points ( ) given as :
8

iii) RT- is an algorithm that takes the points collected from the AOs skeleton images computed from resulting encludian *EDT* images via watershed transformation [29] and determine all lines on which these points lie. The RT from the EDT skeleton image represented through the functionality *R*(*r*,*θ*) is usually defined as a series of line integrals through *EDT*(*x*,*y*) at unique offsets from the origin defined mathematically as:
9

Where, *θ* and *r* are the angle and the perpendicular offset, respectively. Once the projection data are obtained, the average value is used to extract a new feature vector set. The average was then obtained for 180°rotation of angle *θ*. As a result, the new vector point’s *f*_*RT*_ of size 180 × 1 are extracted from each EDT skeleton images and the normalized feature vector  given as:
10

iv) OH- The first step for generating the OH descriptor is to compute the derivatives direction by convolving the gradient mask with the EDT gray scale image. For each *EDT*(*x*,*y*), the OH with different directions *N* ranging from 0 to *π* is computed simply by splitting the histograms into 18 equal dimension bins (*N*/*m*) with size of each bin set to be *m* = 10. The particular OH of all directions are concatenated in vector points (*f*_*OH*_). Two filters *F*_*x*_ and *F*_*y*_ of size (1 × 3) for horizontal and vertical filtering, respectively are convolved with the *EDT*(*x*,*y*) to generate the two gradient images of *R*_*x*_(*x*,*y*) and *R*_*y*_(*x*,*y*) in both directions as follows:
11

In which ∗ denotes the convolution operation and the magnitude *C*(*x*,*y*) calculation given as:
12

Next the gradient *G*(*x*,*y*) is computed by applying:
13

The gradient directions (*x*,*y*) are then measured with respect to the x-axis as:
14

Summation *P*(*x*,*y*) is connected with *α*(*x*,*y*) for every bin (*m* = 10) given as :
15

Once the *P*(*x*,*y*) is obtained for each bin, those values are accumulated in a single vector, where the histogram values are computed simply by counting the angles belonging to the respective bins. The normalized vector points  given as:
16

**b) Contour-based fracture characterization (CB-FC) approach** This section presents the contour-based algorithm as a second approach for fracture characterization. The CB-FC only exploits the shape contour and boundary information, and seeks the possibility of using this information as a key and guideline to differentiate between AO fractures and capture the shape characteristics using Fourier descriptors derived from geometric measurements. In the sagittal view, the shape boundary represented by the 9-APR or B-SR contour includes the superior and inferior points on both anterior and posterior sides of the vertebrae shape. These coordinates of the points are treated as significant to guide in the CB-FC approach consisting of the Global shape profile (GSP) and Shape signature (SS) which are described below:

i) GSP - represents features that are fundamental to a shape. In this work, several shape parameters have been introduced and used to describe the GSP. Usually, the simple geometric characteristics may merely discriminate shapes along with significant dissimilarities. Consequently, there are definitely not appropriate as a stand-alone shape description.
ii) SS - In general, is any one-dimensional function derived from the coordinates of shape boundary or boundaries [30, 31]. This SS generally catches the perceptual feature of the shape [32]. SS can easily identify the shape independently, and is frequently utilised as a pre-processing tool to help additional feature extraction algorithms such as Fourier and wavelet descriptors. The two SS, central distance and complex coordinates (position function), are considered in this work, to extract the Fourier descriptor. The reason for choosing the SS features is that they are generally employed in recent FD implementations and also have been proved to be practical for common shape representation [33] retrieval.

The central distance *CD* function is an SS-based inter-distance across shapes for every single vertex *P* in a polygon as the length of the angle bisector at *P*, calculated from *P* to the center of the shape. The *CD* is indicated through the distance of the boundary points from the centroid (*C*_*x*_,*C*_*y*_) of the shape and is given by
17

The complex coordinate *CC* function is simply the complex number generated through the coordinates associated with the boundary shape points, *P*(*x*_*n*_,*y*_*n*_). To remove the effect of bias, the shifted coordinate functions are used and the *CC* is expressed by *z*(*n*)
18

*FD*_*s*_ tend to be obtained by utilizing Fourier transform on a shape boundary, commonly represented by (*SS*). *FD*_*s*_ on *GSP* and *SS* are examined for shape analysis. To apply (*FD*_*s*_) on a given shape *s*(*t*), t = 0.1,…, L, all shapes are normalized to *N* points and the discrete Fourier transformation of *s*(*t*) is given by:
19

where, the coefficient (*U*_*n*_) is usually called the shape Fourier descriptor, denoted by *FD*_*n*_, n = 0, 1,.., N-1.

#### The proposed architecture (PA) for the retrieval phase

The PA for the retrieval phase involves two approaches, specifically, retrieval via RB-FC and retrieval via CB-FC. The former involves GW, RT, GLCM and OH algorithms whereas the latter involves FDs. Prior to implementing PA a PMCVNN need to be constructed and implemented. The PMCVNN is a predictor model that was developed based on the CVNN classifier with a specific task to model additional features of the archived images and to perform pre-classification. At its simplest form, this is done by storing the pair [*k*,*f*], where *f* is the image features and k is the vertebra class it belongs to. The PMCVNN process is implemented via two different stages as the following:

**a) PMCVNN construction (Off-line stage)** To produce archived data reference that is stored in the relational database, a visual interpretation (VI) analysis is applied to each archived image based on the shape feature outline to perform vertebrae references. Here, the 9-APR technique was used to establish the ground truth and determine the AOs classes and degree of severity through vertebra irregularity description. Therefore, all vertebra images in the collection are processed off-line and this is known as the predictor model construction by employing several different algorithms as discussed in the indexing phase (IP). A multi-layer perceptron using cross validation process were used in constructing the predictor model and this model was named as PMCVNN and assigned with a task to pre-classify vertebra irregularity. PMCVNN was chosen over the former holdout technique (holdout) because of its superiority.

In this work, a k-fold CV method was considered where k was set to 5. The leave one out fold was used during PMCVNN classifier training and testing was done using the remaining data. The classifier model of various configurations were analysed and receiver operating characteristic (ROC) and area under the curve (AUC) measurements are used to evaluate the performance. ROC graph provides a systematic analysis of sensitivity and specificity of the classification results [34]. AUC takes range of [0 1] such that AUC of 0.5 reflects a random test accuracy, while AUC =1 implies perfect test accuracy. Hence, AUC < 0.5 indicates a rejection in the model test [34].

**b) PMCVNN implementation (Online-stage)** The first step of the PMCVNN implementation involves the features generation of the vertebra query (VQ) image and vertebra archived (VA) images which utilize either the retrieval via RB-FC or Retrieval via CB-FC. Details are as given below.

i) Retrieval via RB-FC: This can be done simply by running one of the RB-FC algorithms in each of the respective images using both shape boundary representations. In the simplest form, it stores the pair(*f*, *k*),where *f* is a feature and *k* is the index of the image. The results from the RB-FC employing the GW, GLCM, RT and OH feature extraction algorithms on both 9-APR and B-SR are obtained to uniquely present the vertebrae irregularity shape via N-dimensional feature vectors, in which N denotes the number of features obtained from each algorithm.
ii) Retrieval via CB-FC: In retrieval via the CB-FC approach, the user is only interested with the vertebra particular outline features, where the position, size, and rotation of the shape are not significant. To ensure this approach works well, the query image shape and the reference shape need to be comparable in ways that the shape representation is invariant to translation, rotation, and scale. Shape invariance can be difficult to attain in the spatial domain however it can be effortlessly achieved in the spectral domain through FDs. As a result, all of the shape signatures are invariant to translation and so the corresponding *FD*. Rotation invariant of the *FD* is realized by ignoring the phase information and considers only the magnitude values of the *FD*.

Since both GSPs and centroid distance (*CD*) are real values, only *N/2* different frequencies in the FDs are essential to index the vertebra shape. Scale invariant is next attained by dividing the magnitude values for the first half of the *FD* zero frequency components (DC component). For complex coordinate (*CC*), signatures, after eliminating the first DC component, the remaining components are used to index the shape. Scale normalization is actually attained by simply dividing the magnitude values of all other descriptors over the second descriptor’s magnitude value.

The second step, is the similarity matching (SM) measurement between the VQ image features and those features of identical class (to the VQ image) in the database. The SM used for features retrieved via RB-FC and via CB-FC, uses a variant of the Euclidean distance and city-block method, known as chi-square or weighted Euclidean distance, which is a distance measure between two points with a differential weighting of the space dimensions. If *f*_*q*_ is a feature from the query image and *f*_*i*_ is a feature from the database, then the city-block can be defined by .
20

The following steps are used to conduct the PMCVNN implementation in the proposed retrieval phase

**Input:** query vertebra; **Output:** retrieved images

1. of the Vertebra Query (*VQ*) image.
  1.1 *Select the query vertebrae image.*
  1.2 *Apply MP to the VQ image using a pre-selected representation either 9-APR or B-SR to obtain the VQ shape model.*
  1.3 *Select retrieval approach either via RB-FC or CB-FC.*
  1.4 *Next, perform feature extraction on the VQ image to produce the VQ*_*fq*_*features.*
2. Enrolment process which is to build the vertebra archive (*VA*_*fq*_) of the feature vector sets of all vertebra images in the database.
3. Scoring process: involves PMCVNN execution and SM computation.
  3.1 *Load the best PMCVNN model.*
  3.2 *Identify VQ image.*
  3.3 *Pre-classify the vertebrae archived (VA) images in the datasets*.
  3.4 *If the VQ and VA images are of identical class, compute the SM index else skip SM computation.*
  3.5 *Sort the SM index in descending order based on the pre-classified vertebrae images.*
  3.6 *Return and display the vertebra images with smallest SM index value.*

### Efficacy of the retrieval process

The efficacy of the image retrieval process is evaluated by comparing features of the query image to those of the retrieved images. When the features of the retrieved images are similar to that of the query, the images are labelled as relevant retrieved images. In this work, for retrieval performance evaluation, the term retrieval precision *Pr@M* was used since evaluation was based on a given cut-off rank, i.e only considers the M-topmost results where M = 5, 10, 15 and 20. As such, it is the number of relevant images retrieved (*N*_*R*_) of M-topmost results by a search to a given query divided by the total number of images retrieved (*N*_*T*_) by that search [35]. Mathematically, it is as shown below.
21

Where (*N*_*R*_) is the number of relevant images retrieved; and (*N*_*T*_) is the total number of images retrieved. For quantitative analysis of the overall retrieval performance, the average group score (*A*_*GS*_) is used and it is based on the total *Pr@M* values divided by the respective value of M which is shown in Equation (22).
22

It is very important to clarify that a good retrieval performance of a CBMIR system does not necessarily correlate to the diagnostic accuracy directly [36]. However, it indicates that the CBMIR system is definitely capable of retrieving the images that are visually similar to the query image with regards to extracted features which in turn implies that those features should be highly effective to represent the searched object for a significant diagnosis of AOs based on standard visual appearance [36]. Every AOs image with respect to both 9-APR and B-SR representation from the archived test database has been tested as the query image, where a record of the 20 topmost similar images and its relative similarity scores have been stored in a database.

## Description of the CBMIR system prototype

**Figure 6:**
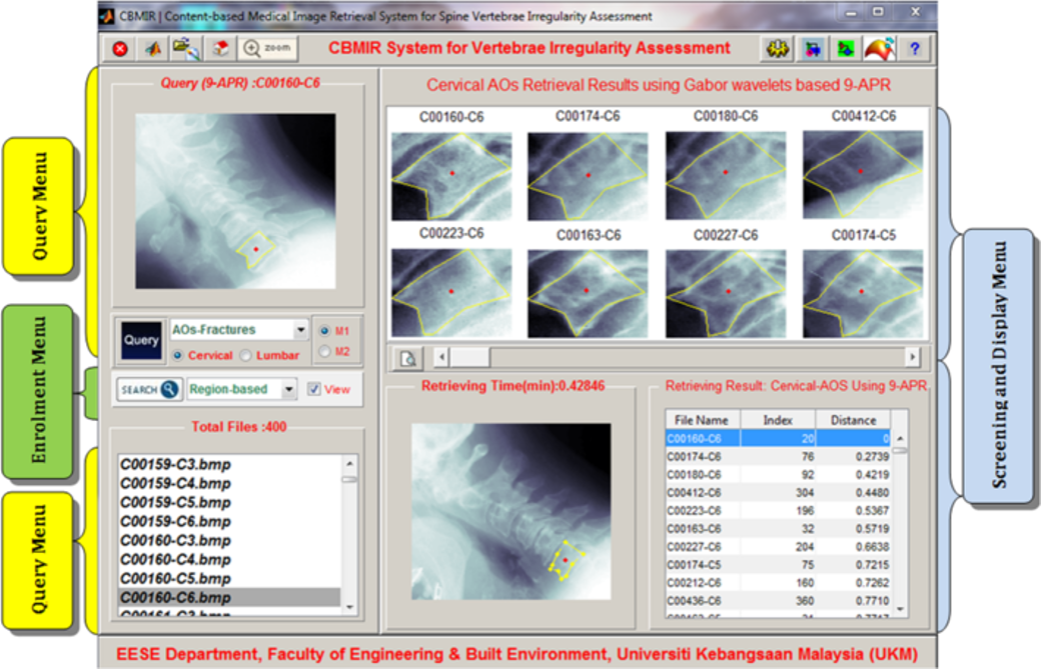
**The Main interface screen shot of the developed CBMIR prototype.** The Query Menu, as labelled on the left by the yellow boxes executes tasks of the query platform. The Enrolment Menu labelled using green colour box was designed to execute tasks of the enrolment platform. The Screening and Display menu as labelled comprises an upper and lower sections that serve to display both visual and statistical retrieval results, respectively. The upper panel on the left/right provides additional functions as zooming, refresh, search, close, etc.

Figure 6 illustrates the main interface screen shot of the developed CBMIR system. It has two main platforms, namely, the query and the enrolment platforms. The former is responsible for acquiring the feature vector that will be presented to the database for matching purposes. Alternatively, the latter is used to retrieve similar images from the database with reference to the query image. Computational algorithms such as GW, GLCM, RT, OH or FDs are used to extract feature for indexing purpose. Finally, all similar images from the same class of the database are ranked and displayed based on the minimum distance similarity measures in ascending order.

### The query menu (QRY-M)

**Figure 7:**
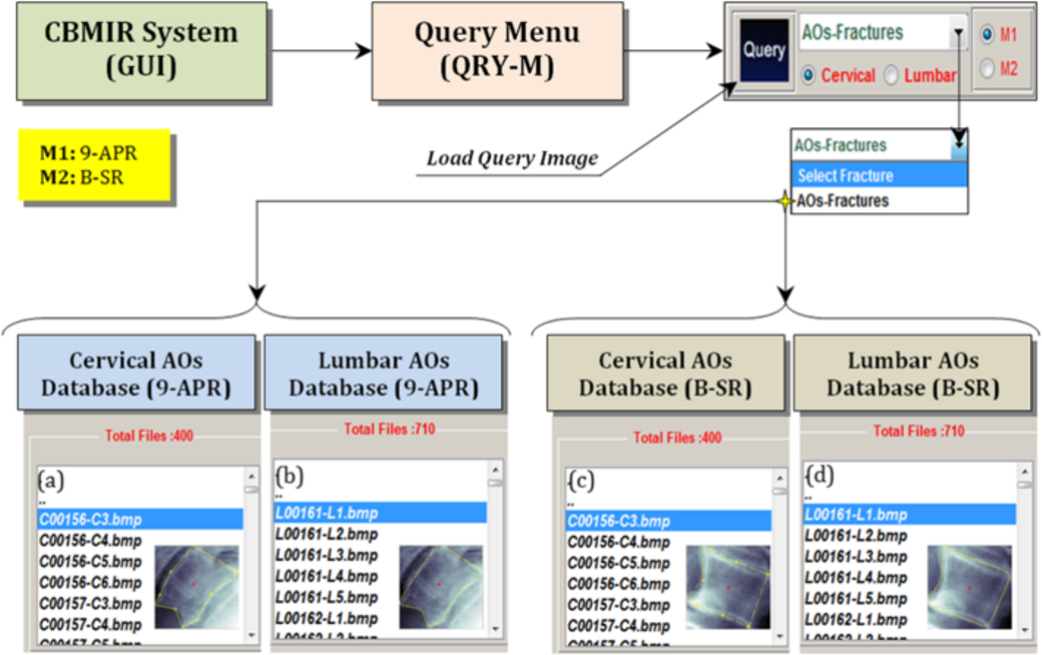
**Presentation of query platform process via QRY-M.** The push-button which is in black is meant for the execution to load the query image (either cervical or lumbar) with the specified fracture AOs represented in two different models 9-APR (M1) and B-SR (M2) as follows: **(a, c)** cervical AOs database with M1 and M2, respectively and **(b, d)** lumbar AOs database with M1 and M2, respectively.

Figure 7 presents the query platform process via the QRY-M. The purpose of the query menu is to generate the query image’s feature vector upon which, the vector will be compared to all existing values stored in the image archive and all closely matched features will be returned. The query image is obtained from the user before it is directed to the following steps where the user is prompted to first select a spine region (either cervical or lumbar), followed by selection of the AOs pathology and finally, choosing the shape boundary representation (either 9-APR or B-SR). The query image by the user will be the basis for the x-ray image comparison of AOs through similarity matching.

### The enrolment menu (ENR-M)

**Figure 8:**
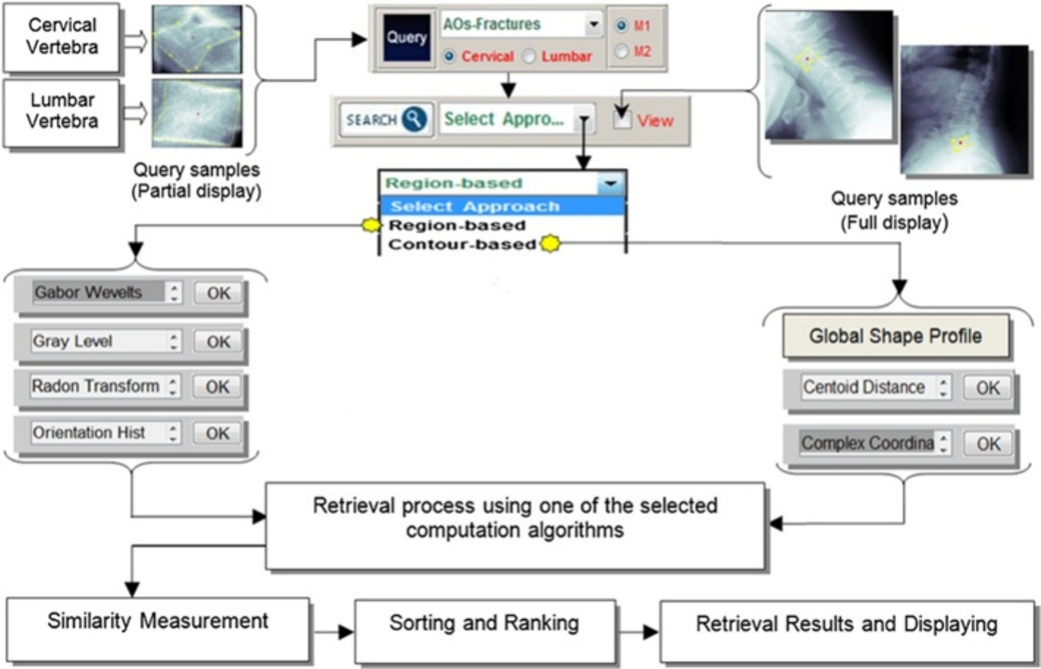
**Presentation of enrolment platform process via ENR-M.** Following a query image selected, search (push-button) for the task to execute the retrieval process and similarity matching (sorting based minimum distance) utilizing the selected computation approach as follows: (i) retrieval via region-based (GW, GLCM, RT and OH), (ii) retrieval via contour-based FDs produced from GSP, CC and CC).

The main purpose of the ENR-M is to display the outputs obtained from the image archive that closely matched the query image based on classification and similarity formulation obtained from the query platform. The ENR-M involved several steps, which are user query on fracture characterization (either Region-based or Contour-based) to extract the query image feature vector, and retrieval process that finds similar images from the annotated database by matching the key attributes associated with the query image. The retrieval process involves user selected feature extraction algorithm to retrieve features in the database and compare it with the resulting query image feature vector by similarity distance measurement. Finally, the most similar images are ranked and returned to the user. Figure 8 illustrates the enrolment platform process via ENR-M.

### The screening and display menu (SD-M)

**Figure 9:**
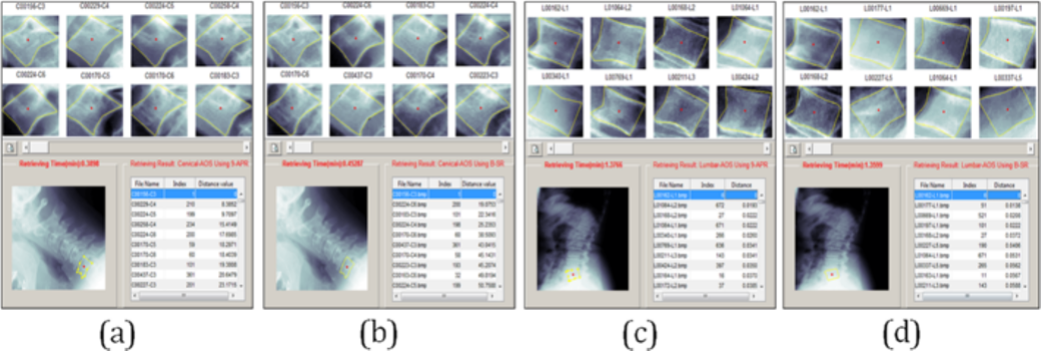
**Sample retrieval results of AOs query using 9-APR via SD-M for cervical and lumbar.** Top of the panel of the screening and display menu visualized the initial top 8 images from the similarity ranking measure (as indicated in the right lower Table) as follows: top retrieved consequence of sample query, **(a)** cervical AOs using 9-APR **(b)** cervical AOs using b-SR **(c)** lumbar AOs using 9-APR and **(d)** lumbar AOs using B-SR. A more retrieval images can be visualized retrieval results by clicking the "slider"? button.

After similar images are ranked and sorted from the enrolment platform, the result and display menu will return the most similar AOs images to the user that includes the following information; the file name, image index, value of the distance being measured and retrieval processing time in seconds. Figure 9 shows the screen shot of the SD-menu for AOs query that were sampled using 9-APR and B-SR for both cervical and lumbar set.

## Results and discussion

### Database description and ground truth establishment

The selected NHANES II (Second National Health and Nutrition Examination Survey) database being utilized in MP is made of 242 digital x-ray images, consisting of 100 cervical and 142 lumbar images. Currently the database is maintained by the Lister Hill National Center for Biomedical Communications, National Library of Medicine (NLM) at the National Institutes of Health (NIH). Table 1 summarizes our selected cases and diagnostic categories of 2220 images from four different AOs image databases for the validation test, which are DB1, DB2, DB3 and DB4. The DB1 and DB2 datasets represent the cervical vertebrae (C3-C6) based on 9-APR and B-SR contour representations, respectively. Meanwhile, the DB3 and DB4 datasets represent the lumbar vertebrae (L1-L5) based on 9-APR and B-SR representations, respectively. In addition, the ground truth for all the different classes of vertebrae irregularities was established manually by marking the points on the vertebrae boundaries by two medical experts to address the issue of inter- and intra-observer variability.

**Table 1:** **AOs vertebrae irregularity datasets and representation techniques used in the study**

	Cervical AOs datasets (C3-C6)	Lumbar AOs datasets (L1-L5)
Representation technique	No of images (Dataset #)	No of images (Dataset #)
9-APR	400 (DB1)	710 (DB3)
B-SR	400 (DB2)	710 (DB4)
Total	**800**	**1420**

The entire AOs datasets were segmented using ASM via 9-APR & B-SR. As mentioned previously, the cervical and lumbar datasets have 10 and 4 different classes, respectively which include the normal case.

### Classification results

**Figure 10:**
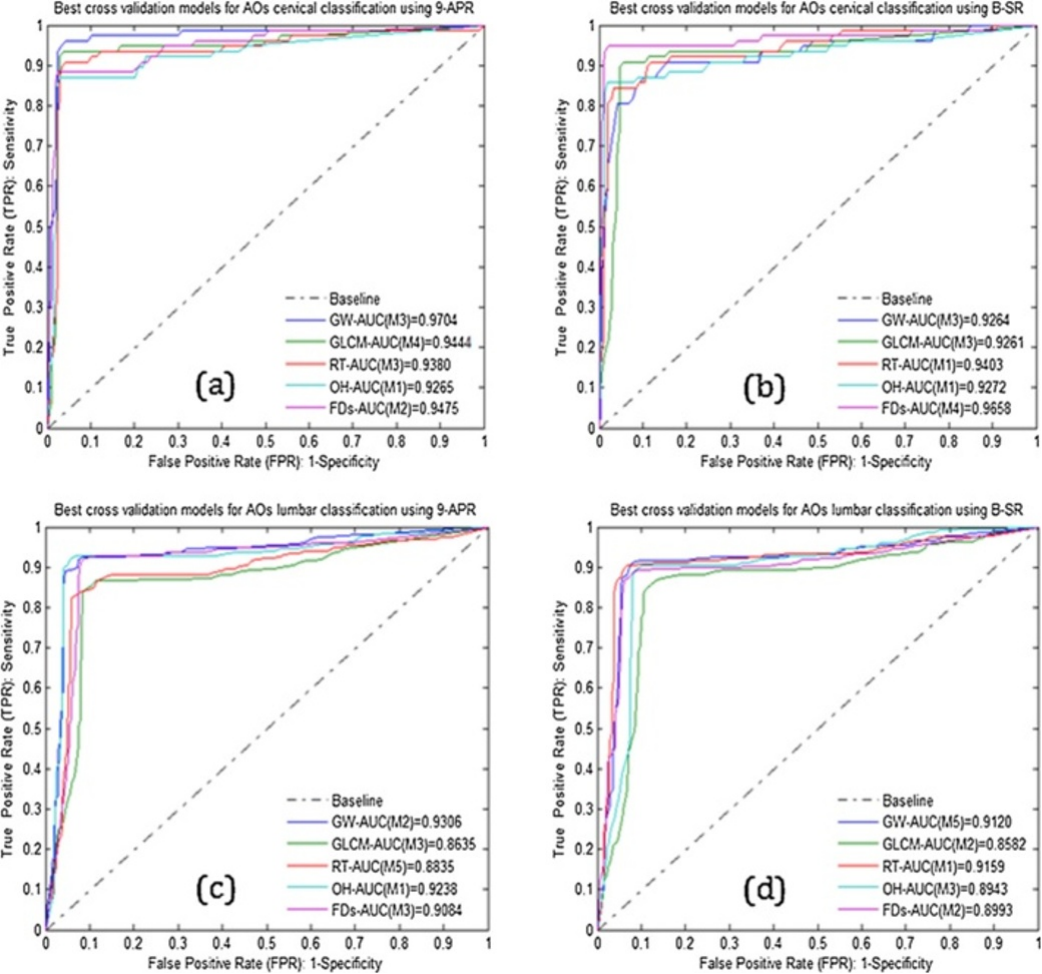
**ROC plots of the PMCVNN classification results of the DB1, DB2, DB3 and DB4 datasets.** Average classification results of the **(a)** DB1 **(b)** DB2 **(c)** DB3 and **(d)** DB4.

In this section, the receiver operating curve (ROC) graph and area under the curve (AUC) score index were used to evaluate the performance of the PMCVNN using k-fold CV (k was set to 5) to model the extracted features for vertebra pre-classification using DB1, DB2, DB3 and DB4 datasets. As previously mentioned, the GW, GLCM, RT, OH and FDs algorithms were implemented to extract the AOs image features which are first normalized and then used as input to the PMCVNN. For training, k-1 fold of feature datasets were used and the remaining is used for testing in each trial and repeated as necessary. Figure 10 (a, b, c and d) show the ROC graphs of the PMCVNN classification performance using the five different features of the GW, GLCM, RT, OH and FDs tested on the DB1, DB2, DB3 and DB4 datasets, respectively where DB1 and DB2 are for the cervical datasets using 9-APR and B-SR, respectively and the remaining for the lumbar datasets of 9-APR and B-SR.The results in Figure 10 suggest the best classification results for cervical and lumbar are obtained using the GW features and 9-APR representation combination with AUC score of 0.9704 and 0.9306, respectively. As such, it can be concluded that the 9-APR technique when combined with GW feature extraction algorithm provide the best combination for the PMCVNN model. As a toolbox, the proposed CBMIR system provides 20 different combination of feature extraction algorithms and vertebrae shape boundary representations with viable performance results of AUC > 0.85 for both of the cervical and lumbar datasets.

### Retrieval results

#### AOs cervical retrieval performance

Tables 2 and 3 are the retrieval results for the various M-topmost retrieved images (for M = 5, 10, 15, 20) relative to the relevant classes of diagnosed AOs from the DB1 and DB2 datasets, respectively using the five feature extraction algorithms (GW, GLCM, RT, OH and FDs) and two different shape boundary representations (9-APR and B-SR) for the standard and proposed CBMIR architectures. Referring to Table 2 for the DB1 (Cervical & 9-APR) dataset, the best retrieval performance for SA was obtained using GW features with an AGs score of 89.16% while for the PA, the best retrieval performance was 96.25% obtained also using GW.

**Table 2:** **Retrieval results for the various M-topmost retrieved images (M = 5, 10, 15, 20) for DB1 dataset (cervical using 9-APR)**

% ***Pr@M*** values for the M-topmost retrieved AOs of DB1 (Cervical & 9-APR) dataset										
	Standard architecture (SA)					Proposed architecture (PA)				
Algorithms	5	10	15	20	***A***_***GS***_	5	10	15	20	***A***_***GS***_
GW	100	100	86.67	70	89.16	100	100	100	85	96.25
GLCM	100	80	73.33	70	80.83	100	90	80.00	70	85.00
OH	80	70	60.00	60	67.50	100	100	93.33	90	95.83
RT	100	80	80.00	75	83.75	80	80	73.33	65	74.58
FDs	100	80	73.33	70	80.83	100	90	80.00	75	86.25
Mean					80.41					87.58

The table tabulates the percent *Pr@M* and *A*_*GS*_ for the SA and PA to show the effectiveness of the proposed CBMIR architecture in performing the retrieval task.

**Table 3:** **Retrieval results for the various M-topmost retrieved images (M = 5, 10, 15, 20) for DB2 dataset (cervical using B-SR)**

% ***Pr@M*** values for the M-topmost retrieved AOs of DB2 (Cervical & B-SR) dataset										
	Standard architecture (SA)					Proposed architecture (PA)				
Algorithms	5	10	15	20	***A***_***GS***_	5	10	15	20	***A***_***GS***_
GW	100	90	73.33	55	79.58	100	100	93.33	65	89.58
GLCM	60	40	33.33	30	40.83	60	60	53.33	50	55.83
OH	100	60	46.67	40	61.66	80	70	66.66	65	70.41
RT	80	60	60.00	50	62.50	100	80	73.33	70	80.83
FDs	80	70	66.67	65	70.41	100	80	70.33	70	80.08
Mean					62.99					75.34

Next, for the DB2 (Cervical & B-SR) dataset of Table 3, the best retrieval performance for the SA and PA were obtained by using the GW features with the score of 79.58% and 89.58%, respectively. As such, it can be deduced that 1) GW feature is the best feature to be used for the retrieval task; 2) the 9-APR shape boundary representation gives better results than B-SR; and more importantly 3) the PA outperformed the SA for all the features of the DB1 and DB2. Additionally, it can be concluded that our proposed CBMIR architecture can significantly improve retrieval performance especially when used with the GW feature extraction and 9-APR shape boundary representation.

#### Visual AOs cervical results

**Figure 11:**
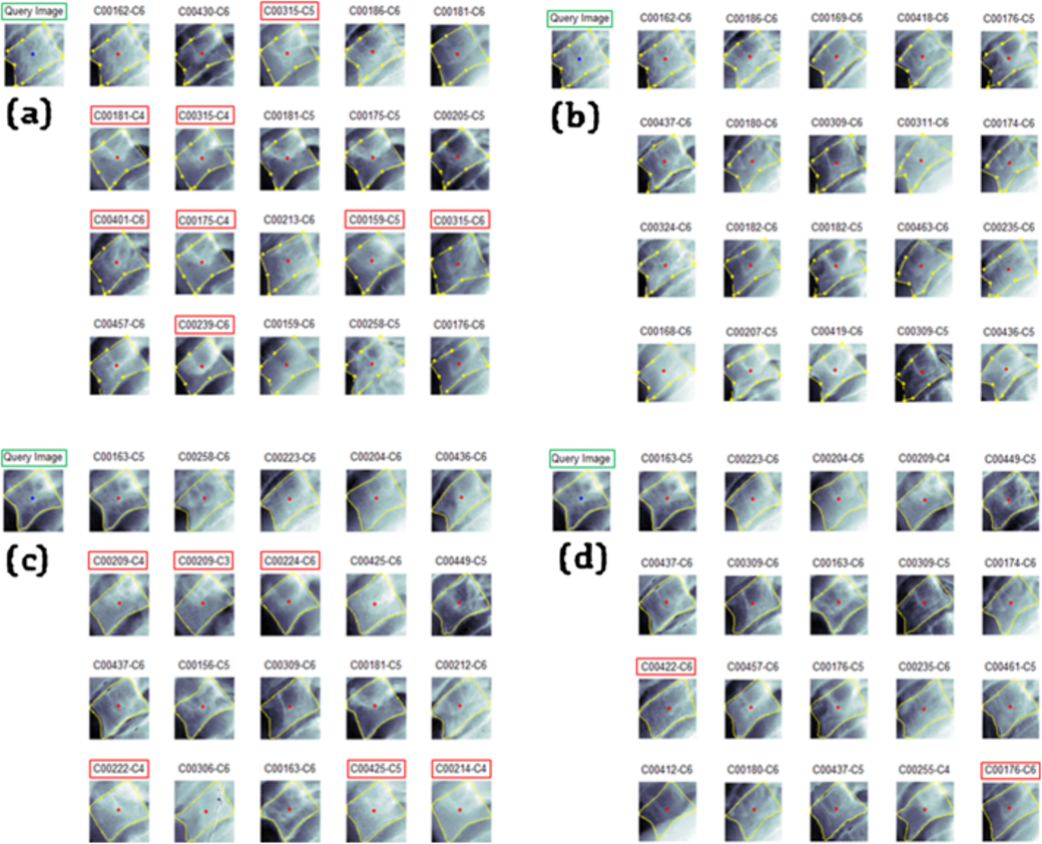
**Retrieval results of the Standard Architecture (SA) and Proposed (PA) using GW features of a selected query image.** Sample results comprising M =20 retrieved images from DB1 (cervical & 9-APR) dataset of a query image for **a)** SA **b)** PA and another set of results from the same query image using DB2 (cervical & B-SR) dataset for **c)** SA and **d)** PA.

Figure 11 depicts the results of the M =20 top retrieved images from a selected query image for the SA and PA using both shape boundary representations of 9-APR and B-SR for the GW feature, which was the best feature in the experiment. As can be seen in Figure 11(b) and (d), the PA retrieved images have fewer error when compared with the SA retrieved images shown in Figure 11(a) and (c). The wrongly retrieved images are labelled with a red box. In brief, these results reaffirmed the earlier quantitative results obtained, thus, it can be deduced that the effectiveness of the proposed CBMIR architecture has been validated.

#### AOs lumbar retrieval performance

Tables 4 and 5 are the retrieval results for the various M-topmost retrieved images (for M = 5, 10, 15, 20) relative to the relevant classes of diagnosed AOs from the DB3 and DB4 datasets, respectively using the same feature extraction algorithms and shape boundary representations as mentioned earlier for the standard and proposed CBMIR architectures. In term of best feature extraction algorithms, the result for the lumbar dataset varied. For DB3 (lumbar & 9-APR) dataset, GW (75%) was noted to be the best feature for the SA where as for the PA, RT (85%) was its best feature. On the other hand, for DB4 (lumbar & B-SR) dataset, the best feature for best retrieval result was GLCM (79.58%) for the SA and OH (88.91%) for the PA. The variation in results for best feature is expected since lumbar shape is more difficult to differentiate than the cervical. Nevertheless, an important point to note from these results is that the PA of the CBMIR system again outperformed the SA.

**Table 4:** **Retrieval results for the various M-topmost retrieved images (M = 5, 10, 15, 20) for DB3 dataset (lumbar using 9-APR)**

% ***Pr@M*** values for the M-topmost retrieved AOs of DB3 (Lumbar & 9-APR) dataset										
	Standard architecture (SA)					Proposed architecture (PA)				
Algorithms	5	10	15	20	***A***_***GS***_	5	10	15	20	***A***_***GS***_
GW	100	80	60.00	60	75.00	100	80	80.00	75	83.75
GLCM	80	70	66.67	65	70.41	100	80	73.33	65	79.58
OH	80	70	60.00	50	65.00	100	80	80.00	75	83.75
RT	80	70	60.67	60	67.66	100	90	80.00	70	85.00
FDs	80	70	60.00	50	65.00	100	80	73.33	70	80.83
Mean					68.61					82.58

**Table 5:** **Retrieval results for the various M-topmost retrieved images (M = 5, 10, 15, 20) for DB4 dataset (lumbar using B-SR)**

	% ***Pr@M*** values for the M-topmost retrieved AOs of DB4 (Lumbar & B-SR) dataset									
	Standard architecture (SA)					Proposed architecture (PA)				
Algorithms	5	10	15	20	***A***_***GS***_	5	10	15	20	***A***_***GS***_
GW	100	70	60.00	60	72.50	80	80	73.33	65	74.58
GLCM	100	80	73.33	65	79.58	100	90	80.00	75	86.25
OH	80	60	60.00	60	65.00	100	80	80.00	75	83.75
RT	100	80	66.67	60	76.66	100	90	80.00	70	85.00
FDs	80	60	53.33	45	59.58	80	70	65.00	60	68.75
Mean					70.66					77.78

#### Visual AOs lumbar results

**Figure 12:**
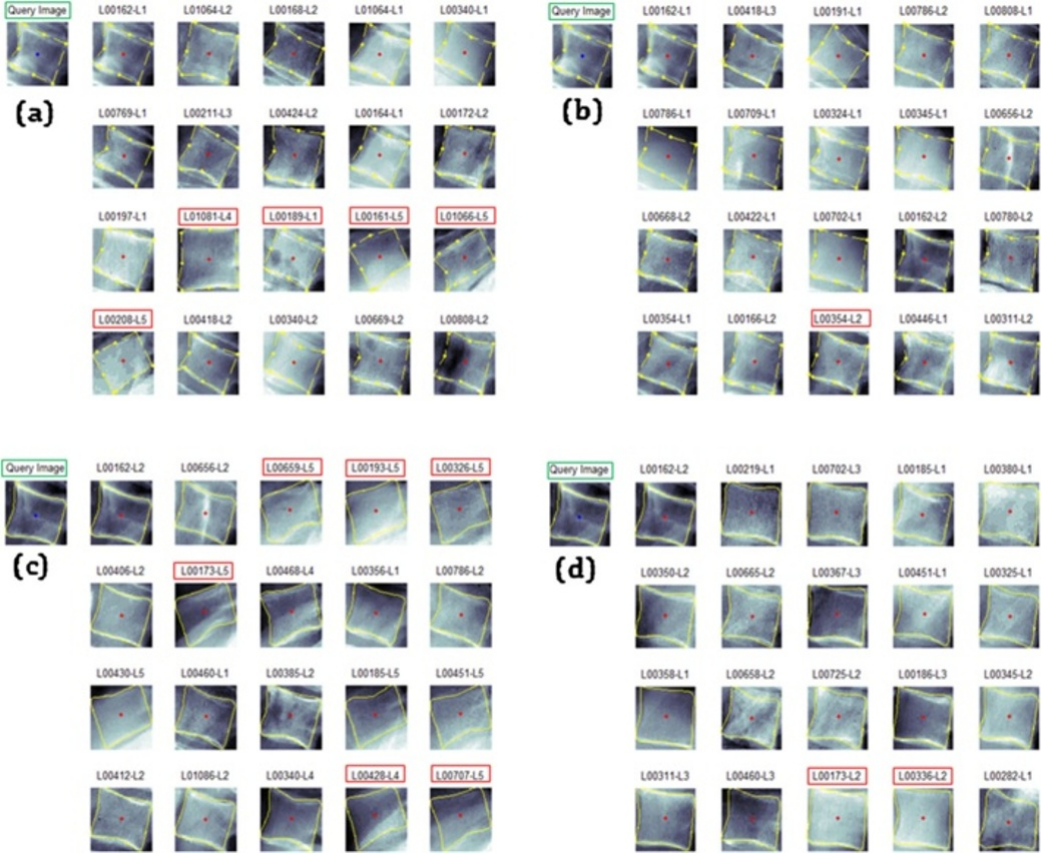
**Retrieval results of the Standard Architecture (SA) and Proposed (PA) using GW features of a selected query image.** Sample results comprising M =20 retrieved images from DB1 (lumbar & 9-APR) dataset of a query image for **a)** SA **b)** PA and another set of results from the same query image using DB2 (lumbar & B-SR) dataset for **c)** SA and **d)** PA.

Figure 12 illustrates the results of the M =20 top retrieved images from a selected query image for the SA and PA using the 9-APR and B-SR for the best feature that yields best retrieval results in the experiment. Again, it was observed that PA retrieved images as shown in Figure 12(b) and (d) have fewer error when compared with the SA retrieved images shown in Figure 12(a) and (c). Similarly, the wrongly retrieved images are labelled with a red box. Again, the PA showed superior retrieval performance than the SA thus, confirm the effectiveness of the proposed CBMIR architecture has been validated.

## Conclusion

CBMIR systems help address the problem that radiologists often miss. For instance, they typically miss signs of vertebrae irregularity that are retrospectively visible in x-ray images. Typically CBMIR systems are proposed to assist radiologist in the assessment of AOs that are under diagnosed by radiologists. In this work, an improvement to the standard CBMIR architecture has been made by modifying the retrieval phase via introduction of the PMCVNN model with the task to pre-classify and matched similar images to the query image. In conclusion, a new CBMIR prototype that provides two options of shape boundary representations, which are the 9-APR and B-SR for vertebrae irregularity was successfully developed. In addition, the new CBMIR prototype also provides several options for extracting features in the IP module to characterize fracture, via two approaches, namely the region and contour based. In sum, the proposed CBMIR prototype not only provide flexible options but it also yields better retrieval results of cervical (*A*_*GS*_ > 87%) and lumbar (*A*_*GS*_ > 82%) when compared to the standard CBMIR system. As a final conclusion, the proposed CBMIR prototype has achieved its initial goal which is to facilitate the medical personnel to carry out visual inspection and perform rapid diagnosis for vertebrae irregularities particularly for the AOs. Further work, is currently under way to improve the results for the Disk Space Narrowing (DSN) vertebrae irregularities and to work on a larger database.

## Abbreviations

CBMIR: Content-based medical image retrieval
ASM: Active shape model
ROI: Regions of interest
RB-FC: Region-based fracture characterization
CB-FC: Contour-based fracture characterization
Pr@M: Retrieval precision at M-topmost results
AG: Average group
AO: Anterior osteoporosis
DSN: Disk space narrowing
SG: Severity grading
L-LHC: Lower left-hand corner
U-LHC: Upper left-hand corner
B-LHC: Both left-hand corner
9-APR: 9-anatomical point representation
B-SR: B-spline representation
GW: Gabor wavelets
GLCM: Gray level co-occurrence metric
RT: Radon transforms
OH: Orientation histogram
GSP: Global shape profile
SS: Shape signatures
CD: Centroid distance
CC: Complex coordinate
FD: Fourier descriptor
MP: Modelling phase
IP: Indexing phase
PA: Proposed Architecture of retrieval phase
SA: Standard Architecture of retrieval phase
R-RB: Retrieval via region-based
R-CB: Retrieval via contour-based
DB: Database.
